# Computed Tomography Perfusion Alberta Stroke Program Early Computed Tomography Score Is Associated with Hemorrhagic Transformation after Acute Cardioembolic Stroke

**DOI:** 10.3389/fneur.2017.00591

**Published:** 2017-11-07

**Authors:** Lan Liu, Bo Wu, Jinglong Zhao, Yanyan Cao, Nikita Dedhia, Louis R. Caplan, Qiaoshu Wang

**Affiliations:** ^1^Department of Neurology, Shanghai General Hospital, Shanghai Jiao Tong University School of Medicine, Shanghai, China; ^2^Center of Cerebrovascular Diseases, Department of Neurology, West China Hospital, Sichuan University, Chengdu, China; ^3^Department of Radiology, Shanghai General Hospital, Shanghai Jiao Tong University School of Medicine, Shanghai, China; ^4^Department of Neurology, University Hospitals of Cleveland, Case Western Reserve University, Cleveland, OH, United States; ^5^Department of Neurology, Beth Israel Deaconess Medical Center, Harvard Medical School, Boston, MA, United States

**Keywords:** cerebral infarction, hemorrhagic transformation, computed tomography perfusion, Alberta Stroke Program Early Computed Tomography scores, cardioembolic stroke

## Abstract

Alberta Stroke Program Early Computed Tomography (CT) score (ASPECTS) has been applied to CT perfusion (CTP) with good interrater agreement to predict early ischemic stroke, and it can be useful in decision making in acute ischemic stroke. The aim of the present study was to assess the predictive value of CTP ASPECTS of hemorrhagic transformation (HT) in acute cardioembolic stroke. This is a single-enter, retrospective study. All patients hospitalized with acute cardioembolic stroke from January 2008 to September 2013 were included. ASPECTS of baseline non-contrast CT, CTP maps of cerebral blood volume (CBV), cerebral blood flow, and mean transit time were collected from 52 consecutive patients with less than 12-h anterior circulation ischemic stroke. MRI scan was performed within 72 h of symptom onset after index stroke including T2*-weighted gradient echo to identify HT. For bleeding risk assessment, CTP and diffusion-weighted imaging ASPECTS were categorized into 0–7 or 8–10. Baseline characteristics, ASPCETS scores and HT were compared. Eighteen (34.6%) patients had HT and four (7.7%) developed symptomatic HT. On univariate analysis, the proportion of patients with CBV-ASPECTS 0–7 was significantly higher in HT patients as compared to patients without HT (44 versus 9%, *P* = 0.005). CBV ASPECTS 0–7 remained independent prognostic factors for HT after adjustment for clinical baseline variables. CBV ASPECTS could be of value to predict HT risk after acute cardioembolic stroke and may be a quick risk assessment approach before reperfusion therapy.

## Introduction

Hemorrhagic transformation (HT) can cause devastating consequence of ischemic stroke, especially for cardioembolic stroke, occurring in up to 90% of patients within the first week after symptom onset (1–4). In recent endovascular therapy for ischemic stroke, computed tomography (CT) perfusion (CTP) was used to identify the ischemic penumbra in the EXTEND-IA trial, and in the MR CLEAN trial, although perfusion imaging was not used as an inclusion/exclusion criteria, CTP was done in about 65% of patients (5, 6). The purpose of using CTP in these studies was to exclude patients with large ischemic stroke and without salvageable ischemic tissue, and such patients have higher odds of hemorrhage or malignant edema caused by reperfusion (7, 8).

Alberta Stroke Program Early CT score (ASPECTS) has been applied to CTP with good interobserver variability, and CTP ASPECTS is more accurate at predicting the extent of reversible and irreversible ischemia than non-contrast CT (NCCT) (9, 10). Diffusion-weighted imaging (DWI) ASPECTS has been proved to be a reliable surrogate of lesion volume in patients with middle cerebral artery stroke, and an independent prognostic factor for symptomatic intracerebral hemorrhage (sICH) after thrombolysis (11, 12). Although automated software analysis can be used to process the CTP imaging, it is not available in many stroke centers. A technique such as ASPECTS could provide rapid, inexpensive and widely applicable assessment of early ischemic changes (EICs). Furthermore, low ASPECTS may be associated with an increased risk of HT. Thus, the present study aims to assess the value of CTP ASPECTS as a method of predicting the HT after acute cardioembolic stroke.

## Materials and Methods

### Participants

We sifted all patients at the Beth Israel Deaconess Medical Center of the Harvard Medical School, Boston who met the international criteria for diagnosis of acute ischemic stroke between January 2008 and September 2013 with the approval of The Institutional Review Board. Etiologic origin of the stroke was determined according to the medical records of the patients using the Causative Classification System for Ischemic Stroke and its electronic implementation available online (https://ccs.mgh.harvard.edu/main.php) (13, 14).

**Figure 1:**
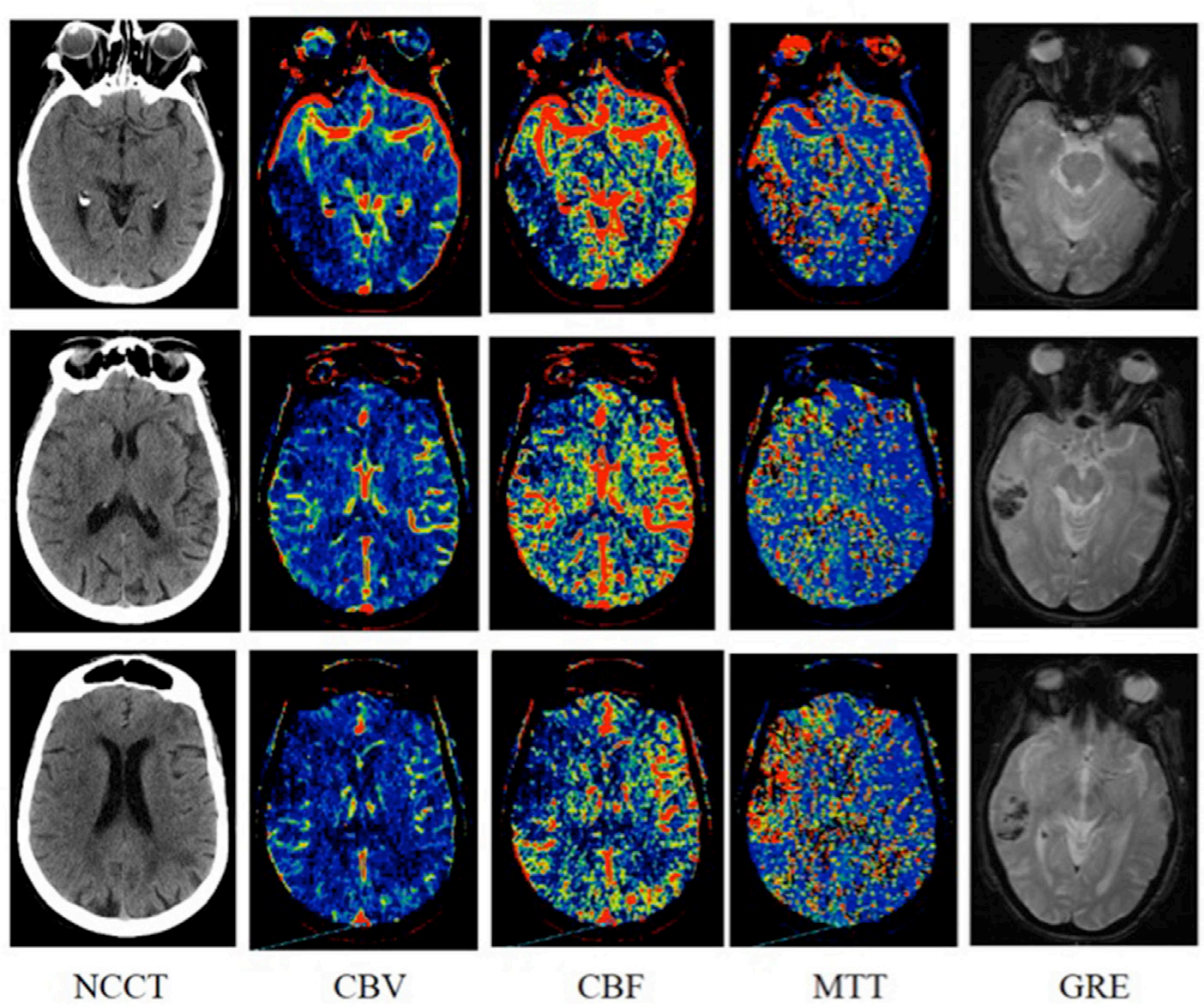
An 87-years-old woman was imaged 3 h after symptom onset with left-sided weakness. Subtle loss of gray-white matter differentiation in the right insular ribbon on non-contrast computed tomography [NCCT; Alberta Stroke Program Early Computed Tomography (CT) score (ASPECTS), 9]. Reduced cerebral blood volume (CBV; ASPECTS, 5) involving the right temporal lobe and frontal lobe in the middle cerebral artery (MCA) territory and more obvious abnormality on cerebral blood flow (CBF; ASPECTS, 4) and mean transit time (MTT; ASPECTS, 4) in the right temporal lobe, frontal lobe and insular cortex. After 7 h of symptom onset hyperintensity on diffusion-weighted imaging (DWI) in the right temporal lobe, frontal lobe, and insular cortex and hypointensity on T2*-weighted gradient echo (GRE) was identified in the right temporal lobe.

Patients were included in the present analysis if (1) they had acute cardioembolic stroke in the anterior circulation and undergone NCCT and CTP within 12 h of symptom onset and (2) an MRI scan was performed within 72 h of symptom onset after index CTP including DWI, T2*-weighted gradient echo (GRE), and MR angiography (MRA). Patients with poor image quality, no GRE sequence, no MRA or CT angiography (CTA), posterior circulation infarction, and anterior cerebral artery infarction were excluded for further analysis. HT was defined as GRE documented hemorrhage in the ischemia occurred within 3 days after treatment onset (Figure 1) (15, 16). Parenchymal hematoma (PH) was defined as hemorrhage with mass effect. sICH definition was PH and NIHSS worsening ≥4 points (17–19). The following clinical variables that have considered to be associated with sICH were collected for each patient: age, NIHSS score on admission, blood pressure level at admission, leukoaraiosis (LA), cerebral microbleeds (CMBs), antiplatelet or anticoagulation use before admission, CHADS2 score, INR level, and reperfusion therapy [including intravenous tissue plasminogen activator (t-PA), intra-arterial delivery of t-PA, and mechanical thrombectomy].

### Imaging

All patients underwent NCCT, CTP, and CTA according to a standardized protocol as part of routine clinical assessment. Whole-brain NCCT was performed using 120 kV, 250 mA, and slice thickness 5 mm. The following CTP used 40 ml non-ionic iodinated contrast at a rate of 4 ml/s *via* a power injector (acquisition time 90 s, 80 kV, 500 mA maximum), providing 16, 5-mm thick slices through the anterior circulation.

MRI scans were performed on 1.5 T scanner. MRI protocol included T1- and T2-weighted, fluid-attenuated inversion recovery (FLAIR), axial trace DWI with 2 *b*-values (0 and 1,000), apparent diffusion coefficient, time of flight MR angiography, and T2*-weighted GRE sequence. The MRI parameters for DWI and GRE were a slice thickness of 5 mm (no gap between slices) with a matrix size of 128 × 128, 24–30 axial slices, and a field-of-view of 240 mm. DWI (EPI spin echo) parameters were a repetition time (TR) of 4,528 ms, an echo time (TE) of 103 ms. Other imaging parameters were as follows: GRE (TR 835 ms; TE 26 ms), FLAIR (TR 9,000 ms; TE 84 ms), T1 (TR 420 ms; TE 8.8 ms), and T2 (TR 4,500 ms; TE 95 ms).

### Image Analysis

All ASPECTS were assessed independently by two experienced investigators, a neuroradiologist (ZJ) and a neurologist (CY) both blinded to patient identity and clinical information. A template of two axial NCCT/CTP/DWI slices with markers for the 10 regions being scores by the NCCT/CTP/DWI ASPECTS was offered. Scoring of NCCT, CTP and DWI was done separately.

Non-contrast CT ASPECTS were accessed for focal parenchymal low attenuation or swelling (Figure 1) (20). All raters evaluated the CTP images in the same order: cerebral blood volume (CBV), cerebral blood flow (CBF), and mean transit time (MTT). The raters scored ASPECTS region as abnormal if there was a relative reduction in CBV or CBF maps as dark blue, or relative increase in MTT maps as red (Figure 1). Relative threshold values for irreversible lesion and penumbra are CBV < 40%, CBF < 30%, and MTT > 145% compared with the normal contralateral hemisphere (5, 9, 21–23). For MRI, relative hyperintensity on DWI ASPECTS region was scored as abnormal (9, 24).

Leukoaraiosis was defined on MR FLAIR image as hyperintense supratentorial white matter lesions. Fazekas scale was used both in periventricular (0 = absent, 1 = caps or pencil lining, 2 = smooth halo, and 3 = irregular periventricular hyperintensities extending into deep white matter) and subcortical areas (0 = absent, 1 = punctuate foci, 2 = beginning confluence of foci, and 3 = large confluent areas) (25, 26). LA was graded in non-ischemic hemisphere and the total Fazekas score was calculated by adding the periventricular and subcortical scores. CMBs were defined as small (generally 2–5 mm in diameter but up to10 mm) areas of signal void on T2*-weighted sequences (27).

To evaluate of the present of vessel occlusion, two observers (Jinglong Zhao and Yanyan Cao) independently reviewed the circle of Willis maximum intensity projection reconstructions of acute CTA and graded middle cerebral artery as proximal and distal occlusion. Follow-up MRA was also graded in the same manner.

### Statistical Analysis

The mean value of ASPECTS of both observers was used for statistical analysis. Non-integral numbers of the mean ASPECTS of the two observers were uprounded for categorization (i.e., if the mean CBV-ASPECTS was 6.5, the patient was categorized in the 7–10 CBV-ASPECTS group). The patients were divided into two groups by the presence of HT at follow up GRE image. First, Mean ASPECTS on NCCT, CTP, and DWI modalities were compared with each other between groups using *t* tests for normal distribution (Kolmogorov–Smirnov test) and the Mann–Whitney *U* for non-normality. Second, ASPECTS were dichotomized (scores 0–7 versus scores 8–10) according to previous CT-based study (11, 28). Then these cut points were used as independent variables in binary logistic regression analysis to determine whether an ASPECTS was an independent prognostic factor for HT. Other independent variables considered in the regression equations were age of 78 years or younger versus older than 78 years, baseline NIHSS of 15 or less versus greater than 15, blood pressure level at admission, Fazekas scale of LA, absence versus presence of acute vessel occlusion, reperfusion therapy, and antiplatelet or anticoagulation use before admission (28). Third, Interobserver reliability was tested for ASPECTS as a continuous variable using an intraclass correlation coefficient with one-way analysis of variance. Results were considered statistically significant at the 5% level. For statistical analysis the SPSS 20.0 software (SPSS Inc.) was used. The weighted kappa statistics and the ROC analyses were calculated with MedCalc (Version 16.8.4) (24).

## Results

Of 61 patients suspected of having an acute cardioembolic stroke in the anterior circulation territory, 9 patients were excluded due to no GRE imaging (1 patient), poor imaging of CTP (3 patients), posterior circulation or anterior cerebral artery infarction (5 patients). Therefore, a total of 52 cardioembolic ischemic stroke patients were included in the analysis (mean age, 75 years; 33 women). The NIHSS score on admission was 10.5 ± 7.0 (interquartile range, 5–16). Median time from symptom onset to NCCT and CTP imaging was 5.2 ± 4.8 h (interquartile range, 2.0–7.0 h) and from symptom onset to MRI imaging was 30.7 ± 30.0 h (interquartile range, 12.0–39.5 h). Of 24 patients who received reperfusion therapy, 15 patients were treated with standard dose intravenous recombinant tissue plasminogen activator (rt-PA). Seven patients received intravenous rt-PA and mechanical thrombectomy, and two patients were given intravenous and intra-arterial rt-PA. The interval time from onset to reperfusion therapy is 2.6 ± 1.0 h (interquartile range, 1.8–3.0 h). Table 1 gives an overview of demographic and clinical variables between HT and non-HT patients. Patients with reperfusion therapy had a 70% risk of HT as compared to a 32% risk in patients without HT. Interobserver reliability for the assessment of ASPECTS was substantial (weighted kappa 0.723 for NCCT, 0.775 for CBV-ASPECTS, 0.778 for CBF-ASPECTS, 0.852 for MTT-ASPECTS, and 0.887 for DWI-ASPECTS).

**Table 1:** Demographic and clinical variables.

Variables	Hemorrhagic transformation		*P*
	Yes (*n* = 18)	No (*n* = 34)	
Sex, male	8 (44)	11 (32)	0.58
Age, >78 years	7 (39)	20 (59)	0.28
NIHSS score > 15	5 (28)	8 (24)	1.00
Systolic blood pressure (mm Hg)	153 (28)	154 (28)	0.93
Diastolic blood pressure (mm Hg)	78 (18)	82 (18)	0.51
Symptom onset to CTP (h)	4.1 (3.2)	5.8 (5.4)	0.50
Symptom onset to MRI (h)	34.2 (34.9)	28.9 (27.4)	0.67
Symptom onset to reperfusion therapy	2.33 (1.0)	2.99 (1.0)	0.42
Antiplatelet use	7 (39)	18 (53)	0.50
Anticoagulant use	3 (17)	7 (21)	1.00
MCA occlusion	15 (83)	24 (71)	0.50
Proximal MCA occlusion	9 (50)	11 (32)	0.34
CHADS score	2 (1)	2 (1)	0.23
Cerebral microbleeds	1 (6)	1 (3)	1.00
INR	1.2 (0.3)	1.4 (0.6)	0.34
Fazekas score	2 (2)	3 (2)	0.70
Reperfusion therapy	13 (72)	11 (32)	0.01

*Values are mean (SD) or *n* (%), as appropriate*.

The mean ASPECTS showed significant difference between modalities in the following order (Table 2): NCCT and CBV > CBF > MTT and DWI. Mean ASPECTS on NCCT and CBV, MTT, and DWI were similar (Table 2). CBV ASPECTS exceeded other modalities especially MTT (mean, 2.5).

**Table 2:** Comparison of the mean value of ASPECTS (95% CI for difference between means, paired *t*-test).

All patients	NCCT	CBV	CBF	MTT	DWI
Mean (SD)	8.6 (1.3)	8.8 (1.7)	7.2 (2.0)	6.3 (1.8)	6.6 (2.0)
NCCT		−0.9–0.4	0.6–2.2^a^	1.5–3.0^a^	1.2–2.7^a^
CBV			1.0–2.2^a^	1.7–3.2^a^	1.6–2.8^a^
CBF				0.3–1.3^a^	−0.2–1.4
MTT					−1.2–0.6

*^a^Bonferroni’s adjustment for multiple t-test comparisons, significant P-value <0.002*.

*ns, not significant; CI, confidence interval; NCCT, non-contrast computed tomography; CBV, cerebral blood volume; CBF, cerebral blood flow; MTT, mean transit time; DWI, diffusion-weighted imaging*.

Eighteen (34.6%) patients had HT. Six patients (11.5%) had parenchymal hemorrhage, and four (7.7%) developed sICH. All the sICH patients were given reperfusion therapy. On univariate analysis, the proportion of patients with CBV-ASPECTS 0–7 was significantly higher in HT patients as compared to patients without HT (44 versus 9%, *P* = 0.005; Table 3). The proportion of patients with DWI-ASPECTS 0–7 was 89% in HT patients and 47% in patients without HT (*P* = 0.008; Table 3). Accordingly, mean DWI-ASPECTS were significant lower in HT patients as compared to patients without HT [6 (2) versus 7 (2), *P* = 0.005; Table 3].

**Table 3:** Comparison of the ASPECTS for HT versus non-HT, using *t*-tests and dichotomous ASPECTS cut point.

ASPCETS	Hemorrhagic transformation		*P*
	Yes (*n* = 18)	No (*n* = 34)	
NCCT, mean value	8 (1)	9 (1)	0.42
NCCT 0–7 vs. >7	4 (22)	8 (24)	1
CBV, mean value	8 (2)	9 (2)	0.29
CBV 0–7 vs. >7	8 (44)	3 (9)	0.005
CBF, mean value	7 (2)	7 (2)	0.4
CBF 0–7 vs. >7	13 (72)	17 (50)	0.21
MTT, mean value	6 (2)	6 (2)	0.89
MTT 0–7 vs. >7	15 (83)	24 (71)	0.45
DWI, mean value	6 (2)	7 (2)	0.005
DWI 0–7 vs. >7	16 (89)	16 (47)	0.008

*Values are mean (SD) or *n* (%), as appropriate*.

*NCCT, non-contrast computed tomography; CBV, cerebral blood volume; CBF, cerebral blood flow; MTT, mean transit time; DWI, diffusion-weighted imaging*.

Sensitivity, specificity, positive predictive value (PPV) and negative predictive value (NPV) for HT prediction are provided in Table 4 by dichotomized imaging criteria of CBV and DWI-ASPECTS 0–7 (versus scores 8–10). Sensitivity was low (0.44) for CBV-ASPECTS 0–7, whereas specificity was high (0.91). Oppositely, high sensitivity (0.89) and low specificity (0.53) were found for DWI-ASPECTS 0–7. PPV (0.72) was medium for CBV-ASPECTS 0–7, and low (0.50) for DWI-ASPECTS 0–7. NPV was medium (0.75) for CBV-ASPECTS 0–7 and high (0.90) for DWI-ASPECTS 0–7. Table 4 provides data on medium (0.72 and 0.68) sensitivity and specificity, low (0.54) PPV and high (0.82) NPV for HT prediction by dichotomized therapy criteria of reperfusion therapy and no use. Using a ROC analysis, the area under the curve did not show significance between CBV-ASPECTS [0.678 (95% CI 0.534–0.801)] and DWI-ASPECTS [0.709 (95% CI 0.567–0.827)], indicating the significant predictive values of the two methods.

**Table 4:** Sensitivity, specificity, positive, and negative predictive values for CBV and DWI-ASPECTS 0–7 and recanalization therapy for HT prediction.

	CBV-ASPECTS 0–7	DWI-ASPECTS 0–7	Reperfusion therapy
Sensitivity	0.44	0.89	0.72
Specificity	0.91	0.53	0.68
PPV	0.72	0.5	0.54
NPV	0.75	0.9	0.82

In a binary logistic regression model, CBV-ASPECTS 0–7 was independently associated with HT when controlling for reperfusion therapy [odds ratio (OR) 10.26 (95% CI 1.83–57.64, *P* = 0.008)]. Likewise, DWI-ASPECTS 0–7 was an independent risk factor in this binary logistic regression model [OR 11.61 (95% CI 1.92–70.29, *P* = 0.008)].

## Discussion

Our results indicate that CBV and DWI ASPECTS 0–7 are associated with HT after cardioembolic ischemic stroke.

The ASPECTS scoring system was initial designed to improve EIC detection on CT scans (28, 29). Although the “goldstandard” for the assessment of EIC is DWI, manual volumetry is time consuming and impractical when quick decision making is needed. On the contrary, ASPECTS DWI scores, a semiquantitative tool of the estimation of the DWI lesion size, have been shown to be of value to apply easily and quickly in the clinical setting. Previous study, performed in patients received thrombolytic treatment, demonstrated that pretreatment DWI ASPECTS 0–7 were associated with sICH and DWI AEPECTS scores correlated well with the DWI lesion volume (11). Furthermore, in accordance with previous study, our series in acute cardioembolic stroke indicates that DWI ASPECTS as a semiquantitative estimate of the DWI lesion size might predict HT with high sensitivity but low specificity. However, the DWI data in our study were acquired 30 h after index stroke. At this point, early decisions about thrombolytic treatment had been made.

In most institutions, CTP is still the preferred choice of rapid diagnosis of hyperacute ischemic stroke by generating maps of CBV, CBF, and MTT. Low CBV the same as DWI, is an indicator of infarction. Several studies explored the predictive value of CBV and showed that lower relative cerebral blood volume and very low cerebral blood volume were more powerful predictor of HT than other parameters of CTP in acute ischemic stroke (30, 31). But all those measurements of CBV need software processing and are time-consuming. The current analysis suggests that CBV ASPECTS 0–7 may predict HT risk in an easy and quick way. In our study, the mean value of NCCT and CBV ASPECTS are similar. In HT group there are more 0–7 CBV ASPECTS patients (44 versus 9%) in comparison with no HT group, and 0–7 NCCT ASPECTS are similar between HT and no HT groups. We think it is because that CBV is more sensitive and specific for identifying irreversible ischemic lesion than NCCT. As Figure 1 showed, CBV has the advantage of detecting ischemic areas after acute infarction over NCCT.

Previous study has showed that CBV ASPECTS closely predicted final infarct in patients with major reperfusion, and CBF and MTT ASPECTS predicted final infarct in patients without major reperfusion (9). In our study, about 50% patients received reperfusion therapy. MTT ASPECTS are similar to DWI ASPECTS and CBV ASPECTS are larger than final infarct ASPECTS, which suggest that most patients did not have major reperfusion, and early lower CBV ASPECTS may reflect large final necrotic area and blood–brain barrier (BBB) breakdown. Our findings demonstrate that CBV ASPECTS 0–7 have low sensitivity and high specificity, medium NPV and PPV. Given the rapid applicability and good interrater agreement, CBV ASPECTS may be favored for quick HT risk evaluation of acute cardioembolic stroke.

In our analysis, the similar ASPECTS of MTT and DWI indicate that most reperfusion therapy is ineffective, which is in accordance with previous study that delayed recanalization after acute cardioembolic stroke is an independent predictor of HT (32). Furthermore, our data show that all the four patients with sICH (7.7%) received reperfusion therapy. The findings support the view that the duration of ischemia is an important determinant in the development of HT and delayed recanalization may cause upstream blood flow passing through impaired downstream BBB. Otherwise, proteolysis of BBB triggered by tPA and mechanical damage to the blood vessel endothelium due to endovascular therapy may also contribute to the HT (33).

Similar to prior studies, we have shown that CBF ASPECTS are approximate to DWI ASPECTS and CBV ASPECTS underestimate DWI volumes (34–36). Since the median time between CTP and DWI scans was 25 h, there was likely little infarct growth in most cases. Based on previous studies, it is hard to make treatment decisions due to the high CTP measurement variability caused by poor contrast-to-noise ratios (34, 36, 37). In those studies, the investigators used semi- or full-automated software to measure CTP lesion volume. Our findings of CTP ASPECTS measurement have shown substantial interrater reliability, which indicate a reliable and easy-to-use tool in making clinical assessment (38).

The main limitation of our study is the retrospective nature of the design. DWI ASPECTS were not performed in the hyperacute stage of stroke, which does not support the feasibility of using DWI ASPECTS in real time. In most institutions CT and CTP are still the first choice of evaluation the ischemia of stroke. Due to the higher sensitivity of CTP for detection of early ischemia as compared to CT, it may therefore be possible that CTP ASPECTS were more reliable than CT ASPECTS. Therefore, the relationship of CBV ASPECTS and HT risk may have been established in the present analysis. Other limitations are the relatively small number of stroke patients, which was a consequence of our strict inclusion criteria.

In conclusion, our study indicates that as a widely applicable and semiquantitative methodology that does not require advanced automated software analysis, CBV ASPECTS could be of value in predicting HT risk after acute cardioembolic ischemic stroke and may be a quick risk assessment approach before reperfusion therapy. Obviously, our findings should be prospectively assessed in a large study.

## Ethics Statement

This is a chart review study approved by The Institutional Review Board of the Beth Israel Deaconess Medical Center of the Harvard Medical School.

## Author Contributions

QW and LC had full access to all of the data in the study and take responsibility for the integrity of the data and the accuracy of the data analysis. Study concept and design: QW and LC. Acquisition of data: LL, QW, YC, JZ, and ND. Analysis and interpretation of data: LL, QW, and BW. Drafting of the manuscript: LL, QW, and LC. Critical revision for important intellectual content: LL, QW, BW, and LC. Study supervision: QW and LC.

## Conflict of Interest Statement

The authors declare that the research was conducted in the absence of any commercial or financial relationships that could be construed as a potential conflict of interest. The reviewer NY and handling editor declared their shared affiliation.
